# Chloral

**Published:** 1871-09

**Authors:** 


### ARTICLE VI.

### *Chloral.*

At the present time perhaps no remedy is more extensively used and more generally discussed than chloral. In the minds of the public an exaggerated estimate of its dangerous capabilities is entertained that is not at all borne out by professional experience. Medical testimony seems to be almost uniform as to its comparative innocency in physiological conditions, and those pathological states in which it has proved dangerous have not been defined, nor has any one, to the best of our knowledge, attempted to generalize these objectionable states. In an interesting article on this subject, in the "American Journal of Insanity," Dr. J. B Andrews has collated a number of cases in which excessive quantities have been administered without fatal consequences, and others in which a comparatively small dose has occasioned death. Dr. Williams, of Baltimore, saw a case of attempted suicide in which 600 grs. had been taken, after remaining eighteen hours, without medical interferance. In the "Chicago Examiner" a case is reported, in which 240 grs. were taken by a lady suffering from neuralgia, who also recovered, after eighteen hours of coma, under the use of galvanism.

Dr. Ludlaw had a case in the Philadelphia Hospital, in which 460 grs. were taken at a dose. "Recovered under proper treatment;" the nature of the treatment is not specified, nor the time of insensibility. In the "English Practitioner two cases are recorded—husband and wife—one of whom took 180 grs. and the other 120 grs. yet both recovered without having experienced any ill effect whatever, after a sleep respectively, of twenty-six and eighteen hours. We have ourselves seen a case in which 240 grs. were taken accidentally without inducing sleep, but without ill effect except a slight delirium. On the other hand, we have seen in a case of softening of the brain in an old scrofulous and epileptic subject, the most alarming effect occasioned by 10 grains.

In the "Pacific Medical Journal" a case is related where a comatose condition was induced by 20 grs., from which the patient recovered without subsequent injury. In the hands of Dr. Reynolds, of London, 50 grs. produced

"dangerous symptoms." Dr. Fuller reports in the "Lancet," a case in which 30 grs. twice "produced alarming symptoms," and another in which the same dose caused death. The "Buffalo Medical Journal" contains an account of a case of death from 400 grs. The "Medical News" has a report of a fatal case "in which the post mortem revealed cerebral congestion." The death of two surgeons is reported in the "London Medical Times and Gazette," one of whom suffered from heart disease, and the other "took by mistake an overdose." The amount is not stated in either case. Dr. Hugh Norris, reports, in the "Lancet," a case in which a patient under treatment for hysteria, complicated with spinal irritation, took a large quantity of this drug in addition to that prescribed by the physician in attendance, amounting in all to "712 grs. in nine days; the last 260 of which were taken in thirty-five hours." One hundred hours after death an autopsy was held, of which the following interesting statement is taken from Dr. Andrews' article:

No odor from decomposition perceptible except in the air displaced from the lungs. There were scarcely any puncta sanguinea in the white portion of the brain, which was very fresh and firm, and little if any fluid in the ventricles. The liver was much enlarged, slightly congestive, and somewhat leathery. The kidneys were enlarged, but not apparently diseased. The heart tissue was somewhat pallid; the ventricles were empty; the auricles partially distended by dark semi-coagulated blood. Stomach not opened; body well nourished; other organs healthy but firm; no decomposition; no ordor of chloroform.

A portion of the lung, liver, heart, kidney and spleen were submitted for analysis. From Mr. Stoddard's report we extract: "The first thing that struck me was the very extraordinary way in which the several portions were preser. ved. Even now, although more than a week has elapsed since death, yet not the slightest sign of decomposition has taken place, nor any unpleasant odor. This doubtless is the effect of chloroform in the tissues."

From the *contents* of the stomach, when mixed with potash and soda, and subjected to distillation, drops of pure chloroform were obtained. This was also found in the liver, but in no other organs. There was no odor of chloroform from any of the tissues of the body, and *the contents of the stomach had no perceptible smell of it till after the addition of alkali.*

Tests for all the poisons that were probable were used without success. Mr. Stoddard gives his opinion from the examination: "There seems no doubt that an excess of chloral must have been taken, and the resultant chloroform was so disseminated through the tissues that they were completely preserved."

As to the mode of death, Mr. Norris adopts the opinion of Dr. B. W. Richardson, "that in such cases dangerous decomposition of the blood may occur before coma is produced, and that the repetition of considerable doses of chloral would be followed by the formation of formiate of soda in the blood, by which its coagulating power would be much diminished; and that in such cases the symptoms would be similar to those induced by loss of blood."

It will be observed "no odor of chloroform" was detected in "any of the tissues of the body, and the contents of the stomach had no perceptible smell of it till after the addition of an alkali." Yet Mr. Stoddard, the chemist, who examined the tissues, it would appear, concludes that the absence of decomposition "is the effect of chloroform in the tissues," resulting from the decomposition of the chloral. This would have been a more logical inference had chloroform been found alone in the stomach, where we might suppose a portion of the very large quantity of chloral taken had remained unabsorbed. But it appears drops of chloroform were not only distilled from the stomach, but "*this* was also found in the liver."

Now, is it, in the first place, likely that so volatile a substance as pure chloroform would have remained one hunpred hours in the liver after death; and in the second place, would the addition of an alkali have been necessary to liberate it if the chloral had already been decomposed in the stomach or blood? or, are we to suppose that the blood had been dealkalized by the excessive quantity of chloral used, and hence the excess of chloral alone remained undecomposed? The blood should have been tested to resolve this question. For ourselves, we utterly disbelieve in the decomposition of chloral into chloroform as its normal physiological mode of action, for these reasons: We do not get the same effect from chloroform administered by the stomach, as we should do if chloral is there decomposed. Chloroform administered by the lungs in ten times the quantity that would result from the decomposition in the blood of a full dose of chloral, does not produce as lasting or as profound an effect. Moreover, the character of their respective effects is, in our judgement, entirely different, and this we think, will be apparent to any one accustomed to the use of both drugs who will carefully note them. We are not, however, prepared to state specifically in what this difference consists. And lastly, the fact brought out in the chemical analysis by Dr. Stoddard, that no trace of chloroform was discovered until such chemicals were used as would decompose the chloral had found its way to the tissues in its normal state.

Prof. N. R. Smith, of Baltimore, in some remarks on this agent, made before one of the medical societies of this city, spoke of a troublesome eruption upon the fingers and hands that he had several times observed from the long continued use of chloral, and expressed the opinion that it is a most dangerous remedy when continued for any length of time; this opinion, however, is not shared by any other physician in Baltimore, so far as we are aware; the views of Prof. Smith meeting with unanimous disapproval in a discussion subsequently had in the Baltimore Medical Association. We find no reference whatever to an effect of this kind in Dr. Andrew's report, though in the N. Y. State Lunatic Asylum, to which he is physician, 90 lbs. have been used since February, 1870. The number of cases prescribed for was 370; the average length of time during which it was given was 39 days to the men, 43 to the women. In a case of melancholia it was given in 20 gr. doses for 257 consecutive nights; in a case of mania 60 grs. were given for 195 nights; in three cases of melancholia and one of acute mania it was given in 20 gr. doses for 221, 255, 207, 257 nights, respectively; in two cases of acute mania and one of melancholia, 40 grs. were given for 236, 222 and 207 nights, respectively, and in many cases for very long periods without ill effect.

Dr. Shew, of the Connecticut Hospital for the Insane, refers to a slight irritation of the eyelids resulting from its continuous use, but we no where else find any reference to any ill effect from its continual use. We must therefore infer that the effect observed by Prof. Smith is an exceedingly rare one, or that it was due to some other cause than the administration of chloral.—*Editorial, Balt. Medical Journal.*

